# Prevalence of Self-Reported Hand Eczema Signs among Healthcare Workers after the Third Wave of COVID-19 Pandemic: A Survey in a Northern Italy Hospital

**DOI:** 10.3390/medicina59061054

**Published:** 2023-05-30

**Authors:** Federica Veronese, Elia Esposto, Chiara Airoldi, Carla Gramaglia, Patrizia Zeppegno, Elisa Zavattaro, Paola Savoia

**Affiliations:** 1 SCDU Dermatologia, AOU Maggiore della Carità, C.so Mazzini 18, 28100 Novara, Italy; federica.veronese@med.uniupo.it (F.V.); espostoelia@gmail.com (E.E.); 2 Department of Translational Medicine, University of Eastern Piedmont, Via Solaroli 17, 28100 Novara, Italy; 3 Department of Health Sciences, University of Eastern Piedmont, Via Solaroli 17, 28100 Novara, Italy

**Keywords:** hand eczema, healthcare workers, COVID-19, pandemic

## Abstract

*Background:* Proper hand hygiene is one of the enhanced preventive measures immediately proposed to avoid the spreading of the severe acute respiratory syndrome coronavirus-2, also known as COVID-19. *Objectives:* The objective of this study was to estimate the prevalence of self-reported hand eczema signs and symptoms among healthcare workers in a Northern Italy University Hospital after the third wave of the COVID-19 pandemic. *Materials and methods:* A cross-sectional study was conducted in June 2021. The hospital workers were invited to complete an online questionnaire through a link sent via institutional e-mail to both health personnel and support staff. *Results:* Eight-hundred and sixty-three subjects completed the questionnaire; 51.1% of them self-reported suffering from at least one hand skin lesion. One-hundred thirty-seven responders declared that they changed their hand hygiene habits, and 88.9% of them carried out these modifications both in occupational and domestic environments. In detail, a change in terms of daily hand washing frequency is reported as follows: before the COVID-19 pandemic, only 27.8% and 10.1% of responders washed their hands 10–20 and 20+ times per day, respectively, while after the pandemic, the percentage increased to 37.8% and 45.8%, respectively. When comparing the health care workers with the administrative staff, we observed a statistically significative difference (*p* = 0.0001) in the daily hand washing frequency among the two groups, with a higher value in health care personnel. Accordingly, a higher prevalence of hand eczema signs (52.8% versus 45.6%) was detected in the healthcare group. *Conclusions:* We underline the potential role of the pandemic in the spread of hand eczema as an occupational disease and the need to implement its prevention.

## 1. Introduction

Proper hand hygiene, together with the use of a protective facial mask, is one of the enhanced preventive measures immediately proposed to avoid the spreading of the severe acute respiratory syndrome coronavirus-2 (SARS-CoV-2), also called coronavirus disease-2019 (COVID-19).

However, during the pandemic, the overzealous use of sanitizers and frequent hand washing have caused an increase in the incidence of hand eczema (HE), in the general population, but above all, among healthcare workers (HCWs) [1,2,3]. Even after the completion of the vaccination campaign, additional sanitizing measures have been used to prevent infection transmission, therefore fostering HE cases. HE is often disabling for the patient and difficult to treat for dermatologists, and it may progress to a frequently relapsing chronic disease, particularly for the risk of sensitization to irritants contained in sanitizers [4].

In this study, we aimed to estimate the prevalence of at least one hand skin lesion of HE among the workers in a Northern Italy University Hospital after the third wave of the COVID-19 pandemic. Moreover, possible differences between healthcare workers (HCWs) and administrative subjects in terms of hand hygiene behaviors were evaluated.

## 2. Materials and Methods

A cross-sectional study was conducted in June 2021. A total of about 3000 persons were invited to participate through a link sent via institutional e-mail to health professions students, residents, and employees (health personnel and support staff) at Maggiore della Carità Hospital, Novara, Italy. Participants who agreed to be enrolled in this study were asked to complete an online questionnaire. All the data were collected anonymously through the Research Electronic Data Capture (Redcap) application, commonly used for research purposes, and validated by the University of Eastern Piedmont (UPO). The study design obtained the required ethical approvals of the Ethics Committee (CE 105/21) of Maggiore della Carità Hospital, Novara.

We included all subjects who had signed up to complete the online survey by giving their consent to the processing of their personal data.

Demographic (gender, age, smoking habit) and occupational (type of work, COVID-19 ward exposure) variables were recorded together with hand hygiene behavior information. In particular, participants were questioned regarding the change in hand hygiene (both in domestic and occupational environments) and the daily frequency of hand washing and hand cream application prior to and after the COVID-19 pandemic. Moreover, information related to possible dermatological diseases, including previous diagnosis of hand dermatitis, atopic disease history (asthma, allergic rhinitis, atopic dermatitis, allergic conjunctivitis), and contact skin allergies were recorded. People were also asked to self-evaluate hand skin lesions in terms of the presence of erythema, desquamation, scaling, vesicles, fissures, oedema, and associated symptoms, such as itching or sleep loss related to the HE problems.

### Statistical Analysis

A descriptive analysis was conducted considering demographic data, clinical signs, and hand hygiene behaviors in the whole sample. Absolute and relative percentage frequencies for the categorical variables, while the mean and standard deviation or median and interquartile range for the numerical ones are reported, as appropriate.

The prevalence of subjects complaining of at least one/three hand skin lesions of HE was calculated, and 95% confidence intervals (95% CIs) are reported. Moreover, a focus on these subjects was made. A separate analysis was then performed comparing the healthcare workers (nurses, medical doctors, social workers in public health service, etc.) with the administrative ones. The differences among the groups were evaluated using the chi-squared/Fisher test and t-test or non-parametric alternatives, as appropriate.

The statistical threshold was set to 0.05 (two-tailed), and all the analyses were performed using the software SAS 9.4.

## 3. Results

### 3.1. Sample Characteristics

Of about 3000 subjects invited to participate, 73% were female, and they were prevalently nurses (53%), medical doctors (18%), and social workers in public health service (17%). Of these, only 863 subjects (28.8%) completed the questionnaire. The demographic and occupational characteristics of the responders are summarized in Table 1; in accordance with the overall sample invited to participate in the study, they were predominantly women (n = 651, 75.4%), and the mean age was 41.70 (SD 12.82; range 19–67 years). The sample was prevalently composed of nurses (n = 295, 34.3%) and physicians (n = 174, 20.2%). During the previous two years, 32.9% (n = 282) of the subjects worked in a COVID-19 ward, for a median time of 4 (IQR 2–8) months.

### 3.2. Comorbidities

Comorbidities are detailed in Table 1. Interestingly, atopic comorbidities were reported in 315 (36.5%) subjects; more in detail, allergic rhinitis represented the most-common comorbidity (n = 201, 23.3%) observed in our sample, followed by atopic dermatitis (n = 102, 11.8%), contact skin allergies (n = 93, 10.8%), and allergic conjunctivitis (n = 84, 9.7%).

### 3.3. Hand Hygiene Habits

Hand hygiene habits and the daily frequency of hand washing were also investigated, with particular attention to changes before and after the COVID-19 pandemic. Here, 717 (83.9%) declared that they changed their hand hygiene habits; 88.9% of them (637/717) made these modifications both in occupational and domestic environments, while 9.5% (68/717) only during work and the remaining 1.1% (8/717) only in the domestic environment. Changes in the daily frequencies of hand washing are detailed in Table 1. Interestingly, before the COVID-19 pandemic, only 238 (27.8%) and 86 (10.1%) responders washed their hands 10–20 and 20+ times per day, respectively; after the pandemic, the number of subjects rose to 324 (37.8%) and 393 (45.8%), respectively. In particular, 674 (78.7%) subjects increased their daily frequencies of hand washing, passing from a low to a high frequency. More details on individual changes are reported also in Supplementary Table S1.

Moreover, we noticed that 422 (49.2%) of the subjects applied emollient hand creams regularly before the pandemic, while after COVID-19, the new users were 222 (25.9%). Among subjects who used hand creams (n = 222 + 422), about 64% (n = 415/644) of our sample applied the cream 1–2 times per day and 23.45% (n = 151/644) 3–4 times.

### 3.4. Self-Reported Skin Lesions

Four-hundred forty-one subjects had at least one self-reported skin lesion, and the prevalence of subjects with mild suspected HE was 51.1% (95% CI 47.71%; 54.49%). Considering the presence of subjects with at least three HE signs, the prevalence was 13.9% (95% CI 10.84; 15.34). Erythema and scaling were present in about a third of the sample (n = 299, 34.7% and n = 265, 30.7%, respectively); fissures were indicated 202 times (23.4%), while vesicles and oedema were less self-reported (n = 56, 6.5% and n = 39, 4.5%, respectively). More details on self-reported skin lesions are reported in Supplementary Tables S2 and S3.

### 3.5. Sample Analysis

A detailed focus was made including only the 441 subjects with suspected HE (at least one sign); the data are reported in Table 2. Here, 227 (51.4%) of these patients referred to an associated itch, which influenced normal daily activities and sleeping in 32.6% (74/227) of cases. Moreover, 19.7% (n = 87/441) of subjects with at least one skin lesion were previously submitted to dermatological examination, and almost all of them applied at least one topical product (n = 429/441, 97.3%), as detailed in Table 2. Furthermore, 168 (38.1%) subjects indicated a previous hand dermatitis; of them, 55 (32.7%) had a diagnosis of dyshidrosis and 12 (7.1%) of psoriasis, while the remaining diagnoses were unspecified. Considering the subjects with previous dermatitis diagnosis (n = 168), in the majority of them (117; 69.6%), it was diagnosed over 1 year prior. Interestingly, in such this category, 69 patients (58%) observed a worsening of their HE during the pandemic.

Generally, subjects believed that the incidence or worsening of the disease was related to the increased frequency of hand washing (n = 91/168, 54.2%) and/or the use of alcohol-based sanitizing gels (129/168, 76.8%).

A further analysis was conducted separately for HCWs (n = 657, 76.1%) and administrative staff. The prevalence of at least one hand eczema sign was higher in HCWs than among the other workers: 52.82% (95% CI) vs. 45.63% (95% CI), but no statistical significance was found (*p*-value 0.0719). Interestingly, the two groups declared that their handwashing habits changed likewise (*p*-value 0.9872) during the last year, even if generally, the HCWs tended to wash their hands more frequently. Notably, before the pandemic, only 3.4% (n = 7) of the office workers washed their hands more than 20 times daily, while for the HCW group, the percentage was significantly higher (12.1%, n = 79) (*p*-value < 0.0001). During the pandemic, the daily hand washing frequency increased: the percentage of HCWs that declared washing their hands >20 times was 51.4% (n = 336), compared to 27.9% (n = 57) for the administrative staff (*p*-value < 0.0001). Generally, creams were used more frequently by HCWs than office workers, even if without statistical differences (new use: 26.2% vs. 25.0%, previous use: 50.8% vs. 44.1%, no use: 23.0% vs. 30.9%) (Table 3).

## 4. Discussion

In the present survey-based study, we analyzed the data collected through web-filled questionnaires concerning HE and daily hygiene habits among a cohort of subjects working in a Northern Italy University Hospital. We detected that the frequency of HCWs’ self-reporting of at least one sign and/or symptom of HE increased from 38.1% in the pre-pandemic period to 51.1% in the post-COVID-19 era.

In the literature, changes in the incidence of various dermatological pathologies have been reported in relation to the COVID-19 pandemic [5,6,7]. Notably, the HE prevalence in the pre-COVID-19 era among HCWs varied between 12% and 50% [8,9,10], while during the COVID-19 era, variations between 50% and 90% have been reported [1,4,8,11,12,13], despite some of these data not being fully comparable due to the different HE detection methods (dermatological evaluation versus self-assessment).

In our cohort, the HE prevalence was in accordance with the literature, although this was a survey-based study, and not all the subjects reporting HE signs/symptoms agreed to a subsequent clinical examination.

Erythema, scaling, and fissures were the majority of symptoms reported by the subjects included in our study, perhaps for the greater simplicity of self-diagnosis. These findings are similar to those reported by Guertler et al., with erythema and scaling being the second and third self-reported signs after dryness [11]. However, these signs recurred more frequently also in cases in which the clinical evaluation was performed by the dermatologist [5], confirming their high frequency. In our cohort, 51.5% of the sample referred to an associated itch, which influenced the normal daily activities and sleeping in 32.6% (74/227) of the cases, underlining the impact of eczematous diseases on the patients’ quality of life [6]. In the study by Guertler, self-reported itching was present only in 28.9% of the sample, and this difference could be explained by a small percentage (<10%) of atopic comorbidities in the considered population [11]. Differently, in our cohort, a 36.5% prevalence of atopy was estimated. On the other hand, Reinholz and coll. previously reported in their study that the HCWs with atopy history had no increased risk of HE when compared to those without atopy, but we also have to consider the limited number in their sample [14].

Regarding hand cream application, 50% of our population used cream before the COVID era, while 64% applied it at least 1–2 times a day after the pandemic, particularly emollient (78.2%) and barrier cream (22.7%). These data are significantly different from those reported in the literature (only 23% of the patients applied hand cream during the COVID era [4,11]); this incongruity can be explained by the fact that, in our sample, the 19.8% of subjects with at least one skin lesion and the 38.1% of those with a past diagnosis of hand dermatitis had already been subjected to a previous dermatological examination.

Among the 168 subjects with previous dermatitis diagnosis, most of them had a disease history of more than a year, and 58% observed a worsening during the COVID-19 pandemic; they associated the new incidence or worsening of the disease with an increased frequency of hand washing (54.2%) and the use of alcohol-based sanitizing gels (76.8%). The exacerbation of HE during the COVID-19 pandemic was also reported by Pourani and coll. in their work based on the self-assessment of HE through a web-based questionnaire in Iran. Hence, they found a very high value of HE exacerbation among the HCWs (94.7%) and the non-HCWs (91.3%) [15]. The interpretation of such findings is complex, as we believe that the timing of the questionnaire administration (May, 2020, during the first wave of COVID-19) and the geographical differences of the populations (Iran versus Italy) have to be evaluated.

Furthermore, in the present study, we separately analyzed the data from HCWs and administrative staff. We found that the prevalence of HE signs was higher for HCWs (52.8% vs. 45.6%), even if not having a statistical significance, possibly because the two groups declared that their hand hygiene habits have changed likewise. A change in handwashing habits, in particular more than 20 hand washes per day, has been previously indicated in the literature as an independent risk factor for the development of HE in the post-COVID-19 era [8,16]. In our sample, we noticed that 88.9% of the participants modified their behaviors, both in occupational and domestic environments, with 78.9% of the subjects having increased the daily frequency of hand washing. In particular, the number of hand washings greater than 20 per day increased in our sample from 10.05% to 45.8% (before and after COVID-19), respectively. In the literature, the percentage varied between 20% and 70% [8,11].

Our study suffered from some limitations: (i) there was a low response rate to the web-based survey (only 863/3000 subjects); (ii) the HE signs were self-reported; (iii) the sample consisted mainly of HCWs, but not all employed in COVID departments; (iv) there were classic risk factors for HE present in our cohort: a high percentage of female gender (75.4%), the winter season for the first two pandemic waves, and a high frequency of atopic dermatitis or atopic comorbidities in the considered population. Some of these factors may have affected the frequency of HE while remaining in line with the literature data. In fact, a prevalence of females was also observed in the populations evaluated by Guertler and Erdem (61.4% and 67.3% of female, respectively) [8,11] and the presence of atopic patients was registered also among other HCW groups [16]. Another possible risk of this investigation was that the symptomatic subjects were more prone to respond to the survey.

Therefore, despite the above-mentioned limitations, we can point out that the relatively low detected frequency of HE compared to other similar studies in the literature might be due to the adoption of preventive measures and dermatological evaluations by our subjects; conversely, Erdem et al. reported that patients with HE used moisturizing creams with a therapeutic intent, increasing their use after the development of eczema, rather than for prevention [8,17,18].

## 5. Conclusions

Considering that, despite the containment of the pandemic waves, hand washing and the use of sanitizing gels remain among the most-used measures to reduce infections, we are strongly convinced of the important role played by the dermatologists underlining the importance of HE prevention, a condition that could become a widespread occupational disease. 

## Figures and Tables

**Table 1:** Demographic, occupational, and health characteristics of responders.

Variable	N (%) N = 863
**Gender**	
Male	212 (24.57)
Female	651 (75.43)
**Age, years**	
Mean (SD)	41.70 (12.82)
Median (Q1–Q3)	43 (29–53)
**Smoking habits**	
No	640 (74.59)
Yes	138 (16.08)
Ex (>6 months)	68 (7.93)
Ex (<6 months)	12 (1.40)
**Occupational sector**	
Nurse	295 (34.26)
Medical doctor	174 (20.21)
Medical/nursing students	107 (12.43)
Social worker in public health service	83 (9.64)
Lab technician	59 (6.85)
Administrative	52 (6.04)
Physiotherapist/speech therapist	27 (3.14)
Obstetrician	17 (1.97)
Other	49 (5.68)
**COVID Ward**	
Yes	282 (32.87)
**Clinical Features**	
**Comorbidities**	
Allergic rhinitis	201 (23.29)
Atopic dermatitis	102 (11.82)
Contact skin allergies	93 (10.78)
Allergic conjunctivitis	84 (9.73)
Asthma	51 (5.91)
**Hand and Hygiene Behaviors**	
Hand hygiene behaviors changes	
Yes	717 (83.86)
**Daily frequency of hand washing before COVID-19**	
<5	164 (19.16)
5–10	368 (42.99)
10–20	238 (27.80)
20+	86 (10.05)
**Daily frequency of hand washing after COVID-19**	
<5	7 (0.82)
5–10	134 (15.62)
10–20	324 (37.76)
20+	393 (45.80)
**Use of hand cream**	
No	213 (24.85)
Yes (previous user)	422 (49.24)
Yes (new user)	222 (25.90)

**Table 2:** Subjects with at least one HE sign.

	N = 441 (%)
**Itch**	
Yes	227 (51.47)
**Itch influencing normal daily activity and sleep** (n = 227) *	
Yes	74 (32.6)
**Previous dermatological examination**	
Yes	87 (19.73)
**At least one topical cure**	
Yes	429 (97.28)
**Type of topical cure**	
Emollient cream	345 (78.23)
Barrier cream	100 (22.68)
Corticosteroid cream alone	37 (8.39)
Corticosteroid/antibiotic	48 (10.88)
**Previous hand dermatitis**	39 (8.84)
Yes	168 (38.10)
**Type of hand dermatitis** (n = 168) **	
Dyshidrosis	55 (32.74)
Psoriasis	12 (7.14)
Other	101 (60.12)
**Timing of hand dermatitis** (n = 168) **	
<6 months	26 (15.48)
1 year	23 (13.69)
>1 year	117 (69.64)
Not specify	2 (1.19)
Possible causes associated with hand dermatitis (n = 168) **	
Increase hand washing	91 (54.17)
Use of alcohol-based sanitizing	129 (76.79)
Job change	3 (1.79)

* Calculated only for subjects with itch; ** calculated only for subjects with hand dermatitis.

**Table 3:** Changes in hand washing and care habits.

	Healthcare Workers N = 657 (%)	Administrative N = 206 (%)	*p*-Value
Yes	546 (83.87)	171 (83.82)	0.9872
**Daily frequency of hand washing before COVID-19**			
<5	100 (15.34)	64 (31.37)	<0.0001
5–10	282 (43.25)	86 (42.16)	
10–20	191 (29.29)	47 (23.04)	
20+	79 (12.12)	7 (3.43)	
**Daily frequency of hand washing after COVID-19**			
<5	4 (0.61)	3 (1.47)	<0.0001
5–10	73 (11.16)	61 (29.90)	
10–20	241 (36.85)	83 (40.69)	
20+	336 (51.38)	57 (27.94)	
**Use of hand cream**			
No	150 (22.97)	63 (30.88)	0.0663
Yes (previous user)	332 (50.84)	90 (44.12)	
Yes (new user)	171 (26.19)	51 (25.00)	

## Data Availability

Data will be made available on request.

## Supplementary Materials

The following Supporting Information can be downloaded at: https://www.mdpi.com/article/10.3390/medicina59061054/s1, Table S1: Daily frequency of hand washing before (horizontally) and after (vertically) the COVID-19 pandemic. Table S2: Frequencies of skin lesions. Table S3: Combination of self-reported HE.
