# Supplementary material for: Prevalence of Self-Reported Hand Eczema Signs among Healthcare Workers after the Third Wave of COVID-19 Pandemic: A Survey in a Northern Italy Hospital
Source: Medicina (Kaunas). 2023 May 30;59(6):1054. doi: 10.3390/medicina59061054 (PMC10301701; doi:10.3390/medicina59061054)
Supplement: Supplementary file 1 [file medicina-59-01054-s001.zip › medicina-2342860-supplementary.pdf]

Table S1. Daily frequency of hand washing before (horizontally) and after (vertically) the COVID-19 pandemic.

		After COVID-19				
		<5	5-10	10-20	20+	**Total**
**Before COVID-19**	**<5**	6	94	52	12	164 (19.16)
	**5-10**	1	37	222	108	368 (42.99)
	**10-20**	0	3	49	186	238 (27.80)
	**20+**	0	0	1	85	86 (10.05)
	**Total**	7 (0.82)	134 (15. 26)	324 (37.76)	393 (45.80)	856

Table S2. Frequencies of skin lesions.

VARIABLE	N (%)
**Signs**	
Erythema	299 (34.65)
Scaling	265 (30.71)
Little vesicles	56 (6.49)
Fissures	202 (23.41)
Oedema	39 (4.52)
**Number of signs**	
0	422 (48.90)
1	172 (19.93)
2	156 (18.08)
3	84 (9.73)
4	20 (2.32)
5	9 (1.04)
**Cumulative number of signs**	
0	422 (48.90)
1+	441 (51.10)
2+	269 (31.17)
3+	113 (13.09)
4+	29 (3.36)
5+	9 (1.04)
Mean (SD)	0.99 (1.20)
Median [Q1-Q3]	1 [0-2]

Table S3. Combination of self-reported HE.

**Signs**		n (%)
0	No	422 (48.9)
1	Only oedema	7 (0.81)
	Only fissures	19 (2.2)
	Only little vesicles	10 (1.16)
	Only scaling	55 (6.37)
	Only erythema	81 (9.39)
2	Fissures+oedema	1 (0.12)
	Little vescicles +fissures	1 (0.12)
	Scaling + oedema	1 (0.12)
	Scaling + fissures	41 (4.75)
	Scaling + litlle vescles	3 (0.35)
	Erythema+oedema	5 (0.58)

	Erythema+fissures	41 (4.75)
	Erythema + little vescicles	5 (0.58)
	Erythema +scaling	58 (6.72)
3	Scaling+fissures+oedema	1 (0.12)
	Scaling+little vesclicles +oedema	1 (0.12)
	Scaling+little vesclicles + fissures	2 (0.23)
	Erythema+fissures+oedema	1 (0.12)
	Erythema+little vescicles + oedema	1 (0.12)
	Erythema+little vescicles + fissures	4 (0.46)
	Erythema+scaling+oedema	1 (0.12)
	Erythema+scaling+fissures	63 (7.3)
	Erythema+scaling+little vescicles	10 (1.16)
4	Erythema+scaling+fissures+oedema	10 (1.16)
	Erythema+scaling+little vescicles+oedema	1 (0.12)
	Erythema+scaling+little vescicles+fissures	9 (1.04)
5	Erythema+scaling+little vescicles+fissures+oedema	9 (1.04)
